# Study on the Propagation Characteristics of Terahertz Waves in Dusty Plasma with a Ceramic Substrate by the Scattering Matrix Method

**DOI:** 10.3390/s21010263

**Published:** 2021-01-03

**Authors:** Qingwen Rao, Guanjun Xu, Pengfei Wang, Zhengqi Zheng

**Affiliations:** 1 Shanghai Key Laboratory of Multidimensional Information Processing, East China Normal University, Shanghai 200241, China; 51191214004@stu.ecnu.edu.cn (Q.R.); zqzheng@ee.ecnu.edu.cn (Z.Z.); 2 Peng Cheng Laboratory, Shenzhen 518052, China; 3 Engineering Center of SHMEC for Space Information and GNSS, East China Normal University, Shanghai 200241, China; 4 School of Information Science and Technology, East China Normal University, Shanghai 200241, China; 5 Shanghai Radio Equipment Research Institute, Shanghai 201100, China; piaobo2000@gmail.com

**Keywords:** terahertz wave, dusty plasma, ceramic substrate, propagation properties, scattering matrix method

## Abstract

The propagation characteristics of terahertz (THz) waves incident vertically into inhomogeneous and collisional dusty plasma with a ceramic substrate are studied using the scattering matrix method (SMM). The effects of the incident wave frequency and plasma parameters, such as the maximal electron density, dust particle density, dust particle radius and collision frequency, on the reflectance and transmittance of THz waves in the dusty plasma are discussed. In addition, the differences of the propagation properties in the dusty plasma, with and without ceramic substrate, are analyzed. Meanwhile, the differences of the propagation properties in dusty plasma and common plasma, respectively, with ceramic substrate are also compared. Simulation results show that the substrate and dust particles have significant influence on the propagation characteristics of THz wave in plasma sheath. Finally, the transmission increases with the increase of electron density, dust density, dust particle radius and collision frequency.

## 1. Introduction

When a hypersonic vehicle re-enters the Earth, a high temperature is generated due to the intense friction between the re-entry vehicle surface and the atmosphere, which results in a plasma flow field that is generated by the ionization of gas on the vehicle surface [1,2,3]. Moreover, the plasma sheath covering the vehicle surface will seriously affect the communication between the ground station and re-entry vehicle [4]. Communication may even be interrupted and result in losing the vehicle, which is referred to as a “blackout problem” [5,6]. To improve the communication performance during the re-entry process, many works on the propagation characteristics of electromagnetic (EM) waves in an inhomogeneous plasma sheath have been carried out and numerous achievements have been reported [7,8,9]. In addition, the materials of the reentry vehicle surface appear to be ablated as a result of the high temperature, which results in many ablative particles being produced [10]. The essence of ablative particles is dust particles. This kind of plasma, composed of electrons, ions, neutral particles and ablation particles, is defined as dusty plasma [11]. Therefore, the transmission of EM wave will be hindered through the collision and absorption of charged particles [12,13]. Consequently, the communication quality between the ground and reentry vehicles is further reduced. To the best of our knowledge, the research on the propagation characteristics of EM waves in dusty plasma is still insufficient, and needs to be further studied.

The study of the physics properties of dusty plasma has attracted much interest since the 1980s [14]. The dynamics of negatively charged dust particles of the magnetized plasma sheath in a fully ionized space plasma has been studied by Baishya and Das [15]. Motie and Bokaeeyan analyzed the dust charging process in the presence of radio frequency discharge on low pressure and fully ionized plasma for weak and strong discharge’s electric field, respectively [16]. Moreover, much research on the propagation and scattering properties of EM waves in dusty plasma has been reported [17,18,19]. Cao et al. used the propagation matrix method to study the propagation characteristics of THz waves in inhomogeneous dusty plasma, and the effects of the incidence angle, the density and radius of the dust particles have been discussed [20]. Though the influences of the dusty plasma parameters on the propagation characteristics have been widely discussed, the differences of the propagation characteristics of THz waves under different plasmas and with different bottom substrates are rarely considered. Wang et al. and Dan et al. compared the differences of the propagation properties of THz waves in dusty plasma and non-dusty plasma. The results demonstrated that the transmittance of non-dusty plasma is larger than that of dusty plasma, which reveals that it is easier for EM waves to penetrate non-dusty plasma [21,22,23]. In addition, Wang et al. analyzed the effects of the transmission and reflection coefficients of THz waves in dusty plasma with different medium parameters [24]. Although numerous results have been achieved for EM wave propagation in dusty plasma, the propagation characteristics of EM waves in dusty plasma under different parameters and with a specified system model need further study. These works are of great significance in the research fields of reentry vehicle monitoring and space communication.

In this paper, the model of dusty plasma with ceramic substrate is established. In addition, THz wave has the characteristics of strong penetration and good directivity. To achieve proper propagation characteristics for the specific application in the dusty plasma, the EM waves were expanded from radio frequency to THz frequency [25]. Therefore, the differences of the propagation properties of THz waves under different plasmas are compared, as well as the model with and without a ceramic substrate is analyzed. Besides, the effects of the propagation characteristics of the dusty plasma parameters, such as the maximal density, dust particle density, dust particles radius, and collision frequency, are further discussed with the scattering matrix method (SMM).

The rest of this paper is organized as follows: In Section 2, the propagation model of EM waves in inhomogeneous and collisional dusty plasma is presented and the SMM is further introduced in detail for the physical model. In Section 3, the propagation characteristics of THz waves in the dusty plasma with and without ceramic substrate is compared, as well as the THz waves in dusty plasma and common plasma is discussed. In addition, the variation of the reflectance and transmittance of dusty plasma versus the incident wave frequency for different values of the maximal electron density, dust particle density, dust particle radius, and collision frequency are analyzed. The conclusions are presented finally in Section 4.

## 2. Propagation Model and Formulas

**Figure 1:**
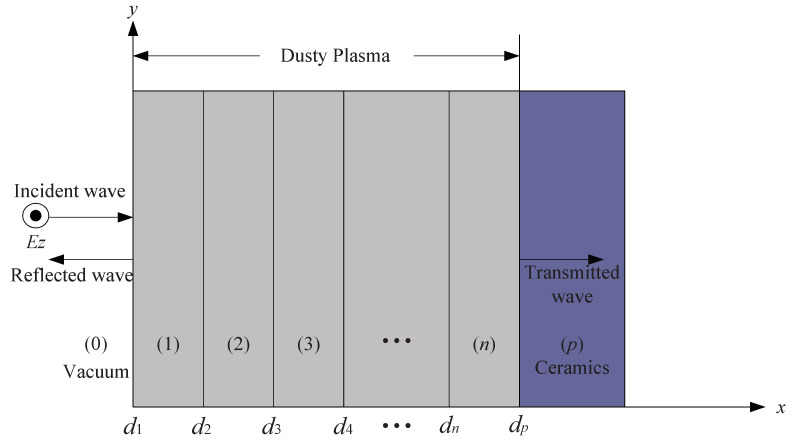
Propagation model of EM wave in inhomogeneous and collisional dusty plasma.

Figure 1 presents the propagation model of the EM wave in inhomogeneous and collisional dusty plasma. According to some recently published literature, the electron density of the whole dusty plasma slab is also considered to be inhomogeneous and obeys the double exponential distribution [21]. The dust particles concentration are set to be uniform in the dusty plasma [17]. In addition, the expression of electron density in shown in Equation (1):

(1) $$N_{e}=\left\{\begin{array}{c} N_{0}e^{x_{1}\left(x-x_{0}\right)},0<x<x_{0}\\ N_{0}e^{x_{2}\left(x_{0}-x\right)},x_{0}<x<L \end{array}\right..$$

As shown in Figure 1, region (0) is the incident region filled with vacuum, region (*p*) is the transmission region filled with ceramic, and between the transmission region and the incident region is dusty plasma. Please note that this model has been studied for the first time to the best of our knowledge. In addition, the dusty plasma is divided into *n* sub-layers, and the electron density in each sub-layer can be regarded as uniformly distributed when *n* is very large. Moreover, the thickness of the whole dusty plasma layer is $d_{p}$ and the interface between the two sub-layers is denoted as $d_{m}$ (*i* = 1, 2, …, *n*, *p*). The thickness of each sub-layer is denoted as $d_{m+1}-d_{m}$.

By solving the Boltzmann and Shukla equations, the complex permittivity of dusty plasma can be expressed as [26]:

(2) $$\begin{array}{cc} \varepsilon_{r}\left(\omega \right) & =1-\frac{{\omega_{pe}}^{2}}{\omega^{2}+{v_{eff}}^{2}}+\frac{c\eta_{ed}\left(v_{ch}+v_{eff}\right)}{\varepsilon_{0}\left(\omega^{2}+{v_{eff}}^{2}\right)\left(\omega^{2}+{v_{ch}}^{2}\right)}\\ \; & +i\frac{1}{\omega }\left[\frac{{\omega_{pe}}^{2}v_{eff}}{\omega^{2}+{v_{eff}}^{2}}+\frac{c\eta_{ed}\left(\omega^{2}-v_{ch}v_{eff}\right)}{\varepsilon_{0}\left(\omega^{2}+{v_{ch}}^{2}\right)\left(\omega^{2}+{v_{eff}}^{2}\right)}\right] \end{array},$$

where *c* is the velocity of light in vacuum, ω is the angular frequency of the incident wave, $\varepsilon_{0}$ is the dielectric constant in vacuum, $\omega_{pe}$ is the angular frequency of plasma, $v_{eff}$ is the collision frequency, $\eta_{ed}$ and $v_{ch}$ are the charge response coefficients and the electron relaxation rate of the dusty plasma, respectively. Please note that $\omega_{pe}$ and $\eta_{ed}$ can be further expressed as [27]:

(3) $$\omega_{pe}=\sqrt{\frac{N_{e}e^{2}}{m_{e}\varepsilon_{0}}},$$

(4) $$\eta_{ed}=\frac{e^{2}\pi {r_{d}}^{2}N_{e}N_{d}}{m_{e}},$$

where $\varepsilon_{0}=8.854\times 10^{-12}$ F/m, the charge of the electron is $e=1.602\times 10^{-19}$ C, the mass of the electron is $m_{e}=9.11\times 10^{-13}$ kg, and $r_{d}$ is the radius of the dust particles. Furthermore, $N_{e}$ and $N_{d}$ are the electron density and dust particle density in the dusty plasma, respectively.

According to Figure 1, the incident and reflected components of the EM wave in region (0) can be expressed by ${E_{z}}^{i}=E_{0}e^{-jk_{0}x}$ and ${E_{z}}^{r}=rE_{0}e^{jk_{0}x}$, respectively. Consequently, the total electric field of the EM wave in the incident region can be written as:

(5) $${E_{z}}^{0}=E_{0}\left(e^{-jk_{0}x}+re^{jk_{0}x}\right),$$

where *r* is the total reflection coefficient, $k_{0}$ is the wave number at the incident region with $k_{0}=\omega /c$.

Similarly, the total electric field of the EM wave in the *m*-th sub-layer and region (*p*) can be expressed as:

(6) $${E_{z}}^{m}=E_{0}\left(b_{m}e^{-jk_{m}x}+c_{m}e^{jk_{m}x}\right),$$

(7) $${E_{z}}^{p}=E_{0}te^{-jk_{p}x},$$

where $k_{m}$ is the wave number at the *m*-th sub-layer, it is a parameter that presents the changing characteristics of EM waves in the media, and $k_{m}=\frac{\omega }{c}\sqrt{\varepsilon_{r}}$. $b_{m}$ and $c_{m}$ are the partial transmission coefficient and the partial reflection coefficient, respectively. Moreover, *t* is the total transmission coefficient, $k_{p}$ is the wave number in the transmission region, $\varepsilon_{p}$ is the relative permittivity of the ceramic substrate, and $\varepsilon_{p}=9.3$. Besides, the thickness of ceramic substrate is 10 mm [28].

The reflection and transmission coefficient of each sub-layer can be obtained through matching the boundary condition of each interface continuously. The total reflection and transmission coefficient can be gained by iterating the scattering matrix equations of each sub-layer. To match the boundary conditions of the interface in the *m*-th sub-layer, we have:

(8) $$\left\{\begin{array}{c} \begin{array}{c} B_{m-1}e^{-jk_{m-1}d_{m}}+C_{m-1}e^{jk_{m-1}d_{m}}\\ =B_{m}e^{-jk_{m}d_{m}}+C_{m}e^{jk_{m}d_{m}} \end{array}\\ \begin{array}{c} B_{m-1}k_{m-1}e^{-jk_{m-1}d_{m}}-C_{m-1}k_{m-1}e^{jk_{m-1}d_{m}}\\ =B_{m}k_{m}e^{-jk_{m}d_{m}}-C_{m}k_{m}e^{jk_{m}d_{m}} \end{array} \end{array}\right..$$

The following matrix form can be obtained by simplifying Equation (8):

(9) $$\left(\begin{array}{c} b_{m}\\ c_{m} \end{array}\right)=S_{m}\left(\begin{array}{c} b_{m-1}\\ c_{m-1} \end{array}\right),$$

where $S_{m}$ is the transfer matrix of the interface in the *m*-th sub-layer:

(10) $$\begin{array}{c} S_{m}={\left(\begin{array}{cc} e^{-jk_{m}d_{m}} & e^{jk_{m}d_{m}}\\ k_{m}e^{-jk_{m}d_{m}} & -k_{m}e^{jk_{m}d_{m}} \end{array}\right)}^{-1}\\ \times \left(\begin{array}{cc} e^{-jk_{m-1}d_{m}} & e^{jk_{m-1}d_{m}}\\ k_{m-1}e^{-jk_{m-1}d_{m}} & -k_{m-1}e^{jk_{m-1}d_{m}} \end{array}\right) \end{array}.$$

Matching the boundary conditions of the incident and transmission regions, the matrix equations of the incident and transmission regions can be written by:

(11) $$\left(\begin{array}{c} b_{1}\\ c_{1} \end{array}\right)=S_{1}\left(\begin{array}{c} r\\ 1 \end{array}\right),$$

(12) $$\left(\begin{array}{c} b_{n}\\ c_{n} \end{array}\right)=S_{p}t,$$

where $S_{1}$ and $S_{p}$ is the transfer matrix of the boundary of the incident and transmission region, respectively. The corresponding equations can be recast as:

(13) $$S_{1}={\left(\begin{array}{cc} 1 & 1\\ k_{1} & -k_{1} \end{array}\right)}^{-1}\left(\begin{array}{cc} 1 & 1\\ -k_{0} & k_{0} \end{array}\right),$$

(14) $$S_{p}={\left(\begin{array}{cc} e^{\left(-jk_{n}d_{p}\right)} & e^{\left(jk_{n}d_{p}\right)}\\ k_{n}e^{\left(-jk_{n}d_{p}\right)} & -k_{n}e^{\left(jk_{n}d_{p}\right)} \end{array}\right)}^{-1}\left(\begin{array}{c} e^{\left(-jk_{p}d_{p}\right)}\\ k_{p}e^{\left(-jk_{p}d_{p}\right)} \end{array}\right).$$

The following equation can be further obtained by recursion of Equations (9) and (11):

(15) $$\left(\begin{array}{c} b_{n}\\ c_{n} \end{array}\right)=S_{g}\left(\begin{array}{c} r\\ 1 \end{array}\right),$$

where $S_{g}$ is the global transfer matrix, and its expression can be written as:

(16) $$S_{g}=\left(\prod_{m=n}^{2}S_{m}\right)S_{1}.$$

Inserting Equation (15) into Equation (12), we have:

(17) $$S_{g}\left(\begin{array}{c} r\\ 1 \end{array}\right)=S_{p}t,$$

Moreover, Equation (17) can be further written as:

(18) $$\left(\begin{array}{cc} S_{g11} & S_{g12}\\ S_{g21} & S_{g22} \end{array}\right)\left(\begin{array}{c} r\\ 1 \end{array}\right)=\left(\begin{array}{c} S_{p1}\\ S_{p2} \end{array}\right)t.$$

The global transfer matrix can be expressed in the form $S_{g}=\left(S_{g1},S_{g2}\right)$, where $S_{g1}$ and $S_{g2}$ represent the first and the second column of $S_{g}$. Substituting $S_{g}$ into Equation (17), the following expression can be obtained:

(19) $$\left(\begin{array}{c} r\\ t \end{array}\right)=-{\left(S_{g1},-S_{p}\right)}^{-1}S_{g2},$$

where *r* and *t* are the total reflection and transmission coefficient, respectively. The expressions of reflectance, transmittance and absorbance can finally be obtained with the following expressions:

(20) $$R={\left|r\right|}^{2},T={\left|t\right|}^{2},A=1-R-T.$$

## 3. Numerical Results and Analysis

**Figure 2:**
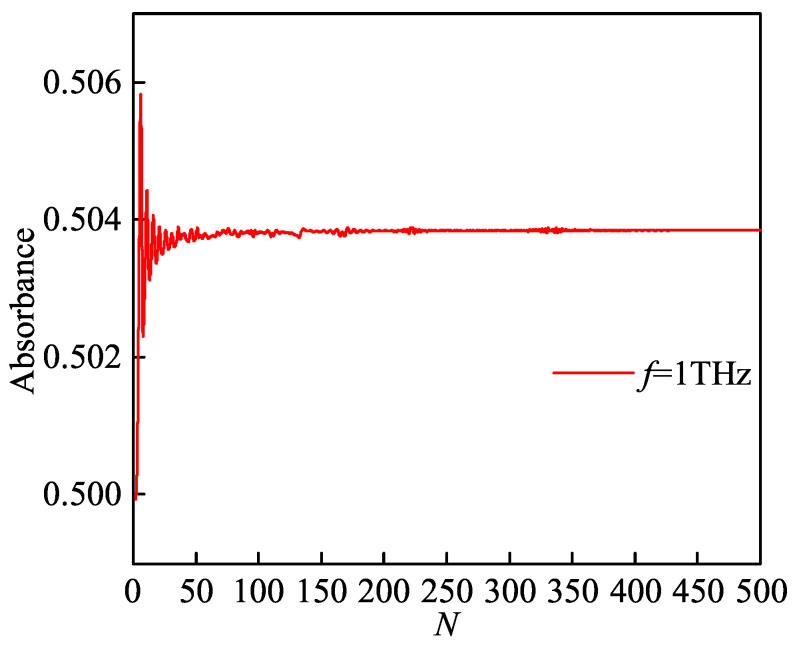
Absorbance versus the number of sub-layer under 1 THz.

Figure 2 presents the absorbance versus the number of sub-layer under 1 THz. In addition, the number of sub-layer is set n=2,…,500. As shown in Figure 2, the absorbance repeatedly vibrates when the number of layers is less than 200, and vice versa. Please note that the simulation time increases with the increase in the sub-layer number since there are many sampling frequency points in the simulation process. Therefore, we set the number of layers to 200 in the following calculations, which can ensure the reliability and accuracy of the results and reduce the time required for simulation.

### 3.1. Effect of the Physical Model with and without Ceramic Substrate

**Figure 3:**
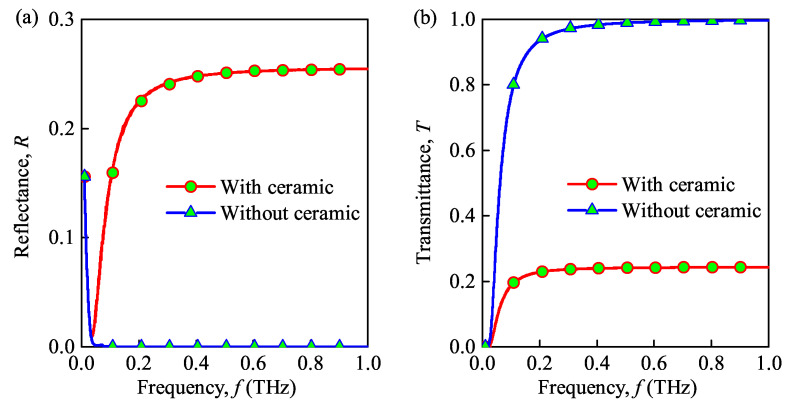
Reflectance (**a**) and transmittance versus (**b**) wave frequency for the case of dusty plasma with and without ceramic substrate.

In this subsection, the reflectance and transmittance of the THz wave in the dusty plasma with and without ceramic substrate are analyzed as shown in Figure 3. The relevant parameters of the plasma are set as follows: the maximal electron density is $N_{0}=1\times 10^{19}$ $m^{-3}$, the dust particle density is $N_{d}=1\times 10^{12}$ $m^{-3}$, the radius of the dust particles is $r_{d}=1\times 10^{-6}$ m, the collision frequency is $v_{eff}=0.1$THz, the number of sub-layers is set as n=200, unless otherwise specified, and the thickness of the dusty plasma slab and the electron relaxation rate are $d_{p}=0.1$m and $v_{ch}=8.7\times 10^{9}$ rad/s, respectively. All of these parameters are selected from some other published literature [12,24].

Please note that the comparison between the two cases is presented in this part. For the first one, only the plasma is considered without any substrate. For the other case, the THz waves are first passing through the plasma slab and then propagate in a ceramic substrate. As shown in Figure 3, the transmittance of the dusty plasma increases with the increase of the wave frequency. In addition, the transmittance of the dusty plasma without substrate is larger than that of the dusty plasma with ceramic substrate, and the reflectance of the dusty plasma without substrate is smaller than that of the dusty plasma with ceramic substrate, which can be attributed to the effect of the ceramic substrate. Besides, the dusty plasma is an important factor that has a great influence on transmittance. Therefore, we conclude that the ceramic substrate has a great influence on the propagation characteristics of THz waves and should be further analyzed for future practical application for the re-entry process.

### 3.2. Effect of Different Plasmas

**Figure 4:**
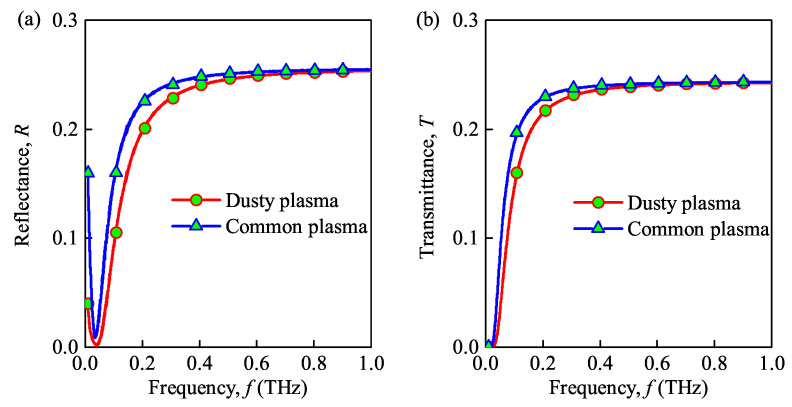
Reflectance (**a**) and transmittance versus wave (**b**) frequency under dusty plasma with ceramic substrate and common plasma with ceramic substrate.

Figure 4 shows the reflectance and transmittance of the THz wave in dusty plasma with ceramic substrate versus various incident wave frequencies. For comparison, the propagation characteristics of the THz wave in the widely analyzed plasma, which is considered to be inhomogeneous, non-magnetized, and collisional, are also given. The maximal electron density and thickness of the plasma are set as $N_{0}=1\times 10^{19}$ $m^{-3}$ and $d_{p}=0.1$ m, respectively. The collision frequency is $v_{eff}=0.1$ THz, Besides, some other parameters of the dusty plasma are $N_{d}=1\times 10^{14}$ $m^{-3}$, $r_{d}=1\times 10^{-6}$ m, and $v_{ch}=8.7\times 10^{9}$ rad/s. From Figure 4a,b, it can be seen that increasing the incident wave frequency increases the transmittance of both the dusty plasma with ceramic substrate and the common plasma with ceramic substrate. Moreover, the transmittance of the common plasma is greater than that of the dusty plasma. This can be attributed to the dust particles of the dusty plasma. Please note that these phenomena are similar to some other studies [21]. This is because there are charged dust particles in dusty plasma, which will collide with electrons, absorb the energy of electromagnetic wave and transfer to neutral particles. Therefore, the transmittance of dusty plasma is lower than common plasma. In addition, the reflectance of the dusty plasma and common plasma first decreases and then increases with the increase of the incident wave frequency. Moreover, the reflectance of the dusty plasma is less than that of the common plasma when the incident wave frequency is very small, but the transmittance and reflectance of dusty plasma are almost equal to those of common plasma in the higher incident wave frequency range (f>0.8 THz).

### 3.3. Effect of Different Parameters of the Dusty Plasma

**Figure 5:**
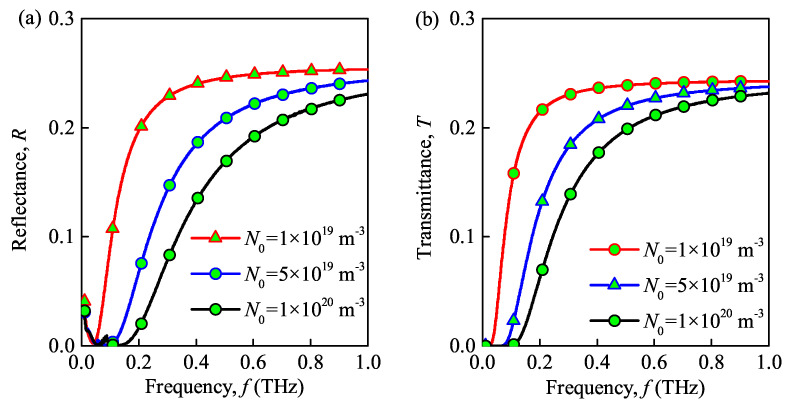
Reflectance (**a**) and transmittance versus (**b**) wave frequency under different maximal electron densities.

**Figure 6:**
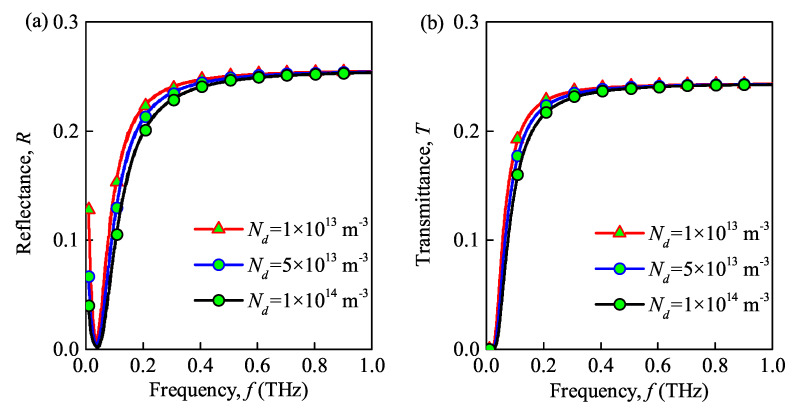
Reflectance (**a**) and transmittance versus (**b**) wave frequency under different dust particle densities.

**Figure 7:**
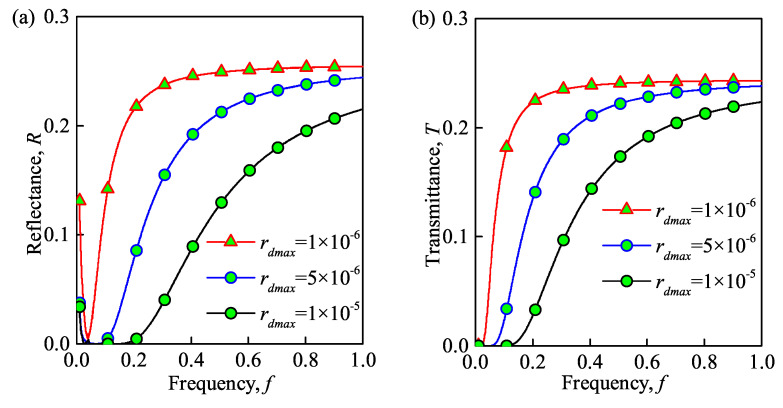
Reflectance (**a**) and transmittance versus (**b**) wave frequency under different radii of dust particles. ($N_{0}=1\times 10^{19}$ $m^{-3}$, $N_{d}=1\times 10^{14}$ $m^{-3}$, $v_{eff}=0.1$ THz).

**Figure 8:**
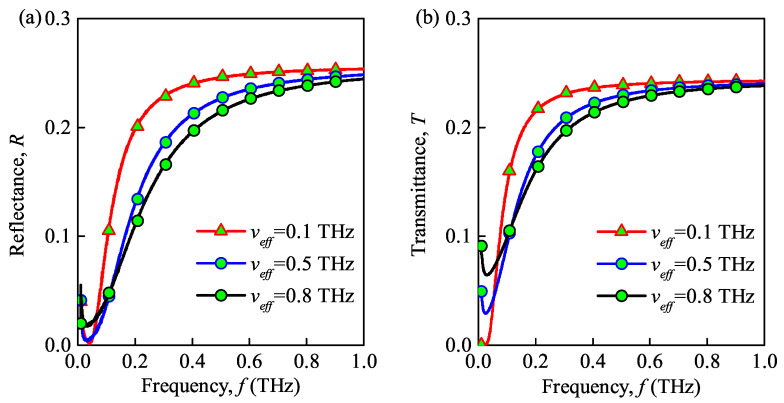
Reflectance (**a**) and transmittance versus (**b**) wave frequency under different collision frequencies. ($N_{0}=1\times 10^{19}$ $m^{-3}$, $N_{d}=1\times 10^{14}$ $m^{-3}$, $r_{d}=1\times 10^{-6}$ m).

The propagation properties of the THz wave in the dusty plasma with ceramic substrate under different values of the maximal electron density, dust particle density, dust particle radius, and collision frequency are shown in Figure 5, Figure 6, Figure 7 and Figure 8. Please note that the maximal electron densities in Figure 5 are $N_{0}=1\times 10^{19}$ $m^{-3}$, $5\times 10^{19}$ $m^{-3}$, $1\times 10^{20}$ $m^{-3}$, the dust particle densities in Figure 6 are $N_{d}=1\times 10^{13}$ $m^{-3}$, $5\times 10^{13}$ $m^{-3}$, $1\times 10^{14}$ $m^{-3}$. In addition, the radii of dust particles in Figure 7 obeys the Gaussian distribution, and $r_{d}=r_{d\max}e^{\left(z_{0}{\left(x-d_{0}\right)}^{2}\right)}$. Besides, the maximal radii of dust are set ad $r_{d\max}=1\times 10^{-6}$ m, $5\times 10^{-6}$ m, $1\times 10^{-5}$ m. Finally, the collision frequencies in Figure 8 are $v_{eff}=0.1$ THz, 0.5 THz, 0.8 THz. Specifically, the other parameters of the propagation characteristics in the dusty plasma slab for the calculation are presented in the label of each figure, and the number of sub-layers is set as n=200.

As shown in Figure 5a, Figure 6a, and Figure 7a increasing the maximal electron density, dust particle density and radius of the dust particles decreases the reflectance of the dusty plasma. These results are similar to the studies in [21,24]. This can be explained by the fact that the energy of the THz wave is absorbed and to the internal energy of the dusty plasma through inelastic collisions, which hinders the transmission of THz waves. Besides, the collision and absorption of charged particles are enhanced as the maximal electron density, dust particle density, and dust particle radius increases. Thus, the absorbance increases and the reflectance decreases in such situation. Moreover, the reflection bandwidth of the THz wave is broadened for larger maximal electron densities and dust particle radii. From Figure 8a, increasing the collision frequency, the transmittance first increases and then decreases. This is because that increasing the collision frequency results in more energy is absorbed from the terahertz waves by the electrons and dust particles and then passed to neutral particles. Similar phenomena also appear in [9]. Therefore, the lower the collision frequency contributes the lower transmittance and the lager absorbance, when f>0.12THz.

The transmittance of dusty plasma versus the incident wave frequency for different maximal electron densities, dust particle densities and radii, and collision frequencies is shown in Figure 5b, Figure 6b, Figure 7b and Figure 8b, respectively. From these figures, the transmittance of dusty plasma decreases with the increase of the electron density, dust particle density, and radius of the dust particles. Moreover, with increasing collision frequency, the transmittance of the dusty plasma is lagerest, when when f>0.12THz. As mentioned above, these phenomena can be explained by the fact that the THz wave energy are absorbed and converted by the collisions between electrons and dust particles, which increases the energy loss of electrons and hinders the transmission of THz waves [29]. In addition, increasing the incident wave frequency increases the transmittance of the dusty plasma. Consequently, the quality of communication between the ground and the re-entry vehicle can be enhanced by increasing the wave frequency. It is noted that the absorbed energy of the THz wave tends to its maximum in the lower frequency range (f≈32 GHz) through the charging of dust particles and the collisions among electrons, neutral particles, and dust particles. Therefore, the dusty plasma behaves as an absorber in this frequency band.

## 4. Conclusions

In this paper, the propagation characteristics of THz waves in dusty plasma with ceramic substrate are studied using the SMM. In addition, the differences of the propagation characteristics of THz waves in dusty plasma with ceramic substrate are compared with some other physical models. The results confirm that the transmittance of the dusty plasma with ceramic substrate is less than that of the dusty plasma without substrate, and the reflectance of the dusty plasma with ceramic substrate is greater than that of the dusty plasma without substrate. Besides, the transmittance and reflectance of the dusty plasma are less than those of the common plasma. Moreover, the variation of reflectance and transmittance of the dusty plasma versus the incident wave frequency for different values of the maximal electron density, dust particle density, dust particle radius, and collision frequency are investigated. These results reveal that increasing the maximal electron density, dust particle density, and particle radius in the dusty plasma decreases the reflectance and transmittance. Moreover, the transmittance and reflectance increase in the lower frequency range and decrease in the higher frequency range with increase of the collision frequency. Finally, the transmittance increases and the reflectance first decreases and then increases with the increase of the wave frequency.

## Data Availability

Not applicable.
